# An In-depth Guide to the Ultrastructural Expansion Microscopy (U-ExM) of *Chlamydomonas reinhardtii*

**DOI:** 10.21769/BioProtoc.4792

**Published:** 2023-09-05

**Authors:** Nikolai Klena, Giovanni Maltinti, Umut Batman, Gaia Pigino, Paul Guichard, Virginie Hamel

**Affiliations:** 1 Human Technopole, Milan, Italy; 2 Department of Molecular and Cellular Biology, Section of Biology, University of Geneva, Geneva, Switzerland

**Keywords:** Expansion microscopy, *Chlamydomonas*, Flagella, Basal body, Super-resolution microscopy, Chloroplast, Membranes, Mitochondria, Microtubules, Cell architecture

## Abstract

Expansion microscopy is an innovative method that enables super-resolution imaging of biological materials using a simple confocal microscope. The principle of this method relies on the physical isotropic expansion of a biological specimen cross-linked to a swellable polymer, stained with antibodies, and imaged. Since its first development, several improved versions of expansion microscopy and adaptations for different types of samples have been produced. Here, we show the application of ultrastructure expansion microscopy (U-ExM) to investigate the 3D organization of the green algae *Chlamydomonas reinhardtii* cellular ultrastructure, with a particular emphasis on the different types of sample fixation that can be used, as well as compatible staining procedures including membranes.

Graphical overview

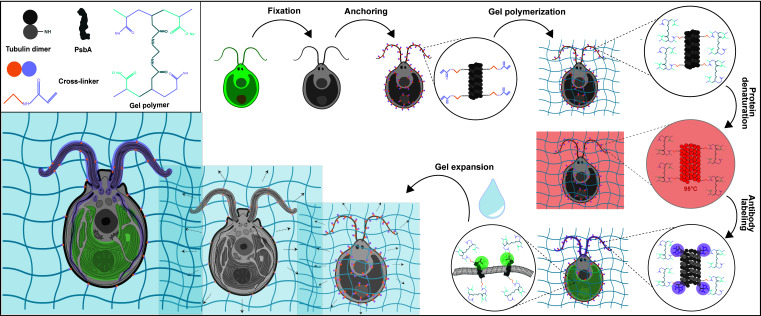

## Background

*Chlamydomonas reinhardtii*, a single-celled green algae, is an ideal model organism in the fields of carbon fixation and ciliary-based motility, owing to its large chloroplast and two flagella. As such, electron microscopists have used *Chlamydomonas* for decades to unravel cellular questions about photosynthetic processes (Salomé and Merchant, 2019), basal body biogenesis and function (Dutcher, 2003), and flagellar motility (Klena and Pigino, 2022). However, the cellular localization of specific proteins of interest is difficult to address by electron microscopy (EM) and, when possible, it requires complex correlative light and EM approaches. Light microscopy and immunofluorescence microscopy are often used to visualize specific protein targets, but the resolution is often insufficient, and the cellular context is indeterminable. Furthermore, utilizing super-resolution techniques such as stochastic optical reconstruction microscopy and stimulated emission depletion requires highly specialized setups and complicated antibody labeling. To circumvent this, we recently utilized super-resolution ultrastructural expansion microscopy (U-ExM) on *Chlamydomonas* to assess how intraflagellar transport trains (IFT) assemble at the ciliary base (van den Hoek et al., 2022). U-ExM is an approach of expansion microscopy derived from the magnified analysis of the proteome protocol that allows for a physical, volumetric expansion of the entire proteome by a factor of 4 (Ku et al., 2016; Gambarotto et al., 2019). By coupling U-ExM with cryo-fixation (Laporte et al., 2022b), NHS-ester (a reactive dye interacting with amines)-mediated pan-ExM experiments (M'Saad and Bewersdorf, 2020), and BODIPY^TM^ (conjugated dye to label lipids) membrane staining (Liffner and Absalon, 2021), the native ultrastructural environment of the entire organism can be assessed through light microscopy, and the localization of specific proteins of interest can be determined by immunofluorescence. The protocol presented here is dedicated to the expansion of *Chlamydomonas* cells but it can also be used for other organisms such as human cells (Laporte et al., 2022a), mouse tissue (Mercey et al., 2022), or yeast (Hinterndorfer et al., 2022).

## Materials and reagents

**Reagents**

***Chlamydomonas*growth media**

1. Tris(hydroxymethyl)aminomethane (TRIS) (Sigma, catalog number: 252859), store at room temperature (RT)
2. Hutner’s trace elements (*Chlamydomonas*Resource Center)
3. Potassium phosphate monobasic (KH_2_PO_4_) (Sigma, catalog number: P5655), store at RT
4. Potassium phosphate dibasic (K_2_HPO_4_) (Sigma, catalog number: P3786), store at RT
5. Acetic acid (Sigma, catalog number: A6283)
6. TRIS-acetate-phosphate (TAP) medium (see Recipes)
7. Phosphate buffer II (see Recipes)
8. Solution A (40×) (see Recipes)

**Regular U-ExM protocol**

1. Formaldehyde, 36.5%–38% (FA) (Sigma, catalog number: F8775), store at RT
2. Acrylamide, 40% (AA) (Sigma, catalog number: A4058), store at 4 °C
3. Sodium dodecyl sulfate (SDS) (Carl Roth, article number: CN30.3), store at RT
4. N,N’-methylenebisacrylamide, 2% (BIS) (Sigma, catalog number: M1533), store at 4 °C
5. Sodium acrylate, 97%–99% (SA) (Sigma, catalog number: 408220), store at -20 °C
6. Ammonium persulfate (APS) (Thermo Fisher, catalog number: 17874), store at RT
7. Tetramethylethylenediamine (TEMED) (Thermo Fisher, catalog number: 171919), store at RT
8. TWEEN 20 (Sigma, catalog number: P1379), store at RT
9. Bovine serum albumin (BSA) (Sigma, catalog number: A3059), store at 4 °C
10. Poly-D-lysine, 0.1 mg/mL (PDL) (Gibco, catalog number: A3890401), store at 4 °C
11. PBS (1× and 10×)
12. ddH_2_O
13. Nuclease-free water (Thermo Fisher Scientific, catalog number: AM9937)
14. Antibodies (primaries and secondaries)
15. NHS-ester Atto488 (Sigma, catalog number: BCCJ6663)
16. NHS-ester 405 (Sigma, catalog number: 250966)
17. NHS-ester Atto 594 (Sigma, catalog number: 08AA12)
18. NHS-ester Atto 647 (Sigma, catalog number: BCCD9924)
19. BODIPY 558/568 (Red) (Thermo Fisher Scientific, catalog number: D3835)
20. Paraformaldehyde (PFA), 16% (EMS, catalog number: 15710)
21. Glutaraldehyde (GA), 25% (Sigma, catalog number: G5882)
22. Methanol (MeOH) (Sigma, catalog number: 34860)
23. α-tubulin antibody (ABCD Antibodies, catalog number: AA345)
24. β-tubulin antibody (ABCD Antibodies, catalog number: AA344)
25. SNAP-tag antibody (New England Biolabs, catalog number: P9310S)
26. PsbA antibody (Agrisera, AS06 143)
27. Centrin antibody clone 20H5 (Millipore, catalog number 04-1624)
28. Formaldehyde/acrylamide mixture (1.4% FA, 2% AA) (see Recipes)
29. Sodium acrylate solution (38% w/w) (see Recipes)
30. Monomer solution, 10 aliquots of 90 μL (see Recipes)
31. 10% TEMED, 10 aliquots of 100 μL (see Recipes)
32. 10% APS, 10 aliquots of 100 μL (see Recipes)
33. Denaturation buffer, pH 9, 100 mL (see Recipes)
34. PBS-Tween (0.1% w/v) (1×, 1 L) (see Recipes)
35. PBS-BSA (2%) (1×, 100 mL) (see Recipes)

**Additional reagents for Cryo-ExM protocol**

1. Acetone (99.8% AcroSeal) (Acros Organics, catalog number: 67-64-1)
2. Ethanol (EtOH, absolute) (Thermo Fisher Scientific, catalog number: 397691000)
3. Dry ice
4. Ethane gas bottle (PanGas). It is also possible to use a 37%:63% mixture of ethane:propane (PanGas), which avoids the freezing of the gas during the preparation

**Materials**

**Regular U-ExM protocol**

1. Erlenmeyer flasks
2. Tweezer Dumont Style 5 with super thin tips (0203-L5-PO) or Negative-Action tweezer Style no. 5 (0203-N5-PO)
3. 12 mm coverslips, high precision no. 1.5H (Marienfeld Laboratory Glassware, catalog number: 47442)
4. 24 mm coverslips, high precision no. 1.5H (Marienfeld Laboratory Glassware, catalog number: 48639)
5. 12-well plates (Thermo Fisher Scientific, catalog number: 150200)
6. 6-well plates (Thermo Fisher Scientific, catalog number: 150239)
7. P1000 pipette tips
8. P200 pipette tips
9. P20 pipette tips
10. Spatula (Bochem, catalog number: 3101)
11. Spoon (Bochem, catalog number: 3421)
12. Razor blade (Carl-Roth, catalog number: CK07.2)
13. 250 mL beaker (VWR, catalog number: 213-1124)
14. 500 mL beaker (VWR, catalog number: 213-1126)
15. Caliper
16. Fine-mesh net (such as mosquito netting or insect netting for a garden)
17. Parafilm (Bemis^TM^, catalog number: PM996)
18. Scissors
19. Pasteur pipettes (Alpha Laboratories, catalog number: LW4111)
20. 1.5 mL Eppendorf tubes
21. 35 mm imaging chamber (metallic O-ring 35 mm) (Okolab, catalog number: RA-35-18 2000–06)
22. Coverslip rack (Thermo Fisher Scientific, catalog number: C14784)

**Additional material for Cryo-ExM protocol**

1. Polystyrene box (23 cm × 23 cm × 21 cm)
2. 5 mL Eppendorf tubes (Eppendorf, catalog number: 0030119401)
3. Tweezer Dumont Style L5 Inox 8 with clamping ring (Electron Microscopy Sciences)
4. Whatman filter paper, 55 mm (GE Healthcare, catalog number: 1001-055)

## Equipment

**Regular U-ExM protocol**

1. 37 °C incubator or climate-controlled room
2. Heat block (Cole-Parmer^TM^, catalog number: SBH130DC)
3. Inverted fluorescent microscope, such as widefield Leica Thunder, SP8, or Zeiss LSM 980, equipped with a 63× oil objective.

**Additional equipment for Cryo-ExM protocol**

1. Cryo-EM manual plunger [both devices used in the preparation of this manuscript were homemade plunging devices; however, commercial manual cryo-plunging devices are usable (https://www.mitegen.com/product/manual-plunge-cooler/), and homemade manual plunging devices can be produced by groups (Comolli et al., 2012)].

## Software

1. Fiji (ImageJ,https://imagej.net/software/fiji/) (Schindelin et al., 2012)
  Depending on the brand of microscope used for imaging, dedicated software is required, such as:
2. LAS X (Leica,https://www.leica-microsystems.com/products/microscope-software/p/leica-las-x-ls/)
3. Zen Blue (Zeiss,https://www.zeiss.com/microscopy/en/products/software/zeiss-zen.html)

## Procedure

- ***Chlamydomonas* cell culture and strains**
  1. Inoculate *Chlamydomonas* strain in at least 10 mL of 1× TAP medium and incubate at 23 °C while shaking (Hoober, 1989). Only a few hundred microliters of culture are required for seeding coverslips.
  2. Grow cells until they reach the logarithmic growth phase and the culture turns bright green. This time corresponds to approximately three days.
*Note: Unlike yeast (Hinterndorfer et al., 2022), the cell wall of Chlamydomonas does not appear to prevent expansion or induce expansion artifacts (Figure 1). We tested two strains, CC124- (cell wall–positive) and CW15- (cell wall–negative), in U-ExM without fixation, and in both cases the cells expand properly by a factor of 4.*
    **Figure 1.**
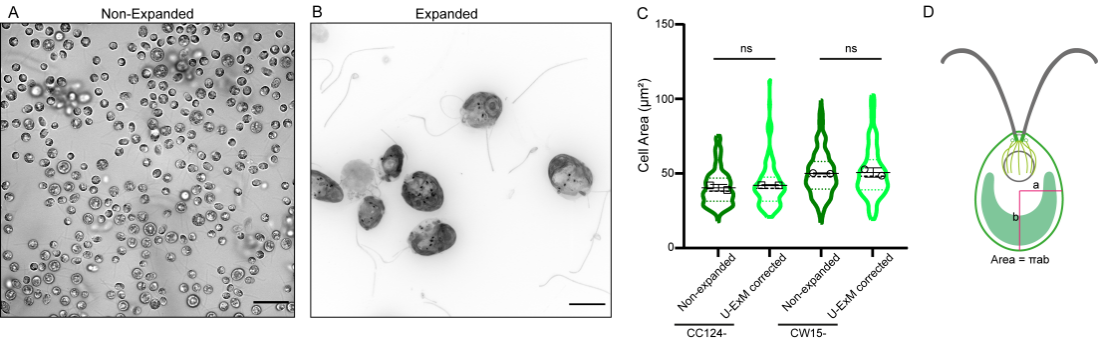
The cell wall of *Chlamydomonas reinhardtii* does not affect the four-fold expansion factor in ultrastructure expansion microscopy (U-ExM). (A) Representative brightfield image of *Chlamydomonas* CC124- (cell wall–positive) strain before expansion, displayed as single z plane. Scale bar: 25 μm. (B) Representative widefield image of *Chlamydomonas* CC124- strain after expansion, stained with pan NHS-ester (far red). The cells are not fixed prior to expansion. Scale bar: 25 μm. The scale bar shows the measured physical length that was not rescaled based on the expansion factor. (C) Measurements of the average cell area of the entire *Chlamydomonas* CC124- and CW15- (cell wall–negative) strains, before and after the expansion. The area of expanded cells is divided by the expansion factor for comparison to the non-expanded cell area. (n = 329, 126, 256, and 111 cells, respectively, from two independent experiments. CC124- non-expanded = 40.50 ± 2.21 μm^2^, expanded = 42.12 ± 0.06 μm^2^, CW15- non-expanded = 50.10 ± 0.36 μm^2^ and expanded = 50.69 ± 3.07 μm^2^; mean ± S.D.) (ns, p > 0.05) (D) Schematic representation of cell area calculation of *Chlamydomonas*.
- **Media, stock solution, and expansion microscopy reagent preparation**
  1. In the days awaiting *Chlamydomonas* growth, prepare stock solutions of sodium acrylate, APS and TEMED aliquots, denaturation buffer, PBS-Tween, and PBS-BSA.
  2. Prepare 12 and 24 mm PDL-coated coverslips by pipetting stock PDL onto coverslips until the surface is covered, which corresponds to roughly 50 μL for 12 mm coverslips and 100 μL for 24 mm coverslips. Place coverslips at 37 °C at ambient humidity, for 30 min, and wash 3× in sterile water. Remove excess water and let air dry. Store coverslips at 4 °C until use.
*Note: Plasma-cleaning the surface of 24 mm coverslips prior to PDL-coating greatly reduces sample drift during the acquisition. See Procedure section E and Notes.*
- ***Chlamydomonas* coverslip seeding, fixation, and gel formation (day 0)**
  1. When *Chlamydomonas* cells reach logarithmic growth, remove PDL-coated 12 mm coverslips from 4 °C and bring to RT for approximately 10 min.
    Using a P1000 with a cut tip, add 100 μL of *Chlamydomonas* culture to the center of the PDL-coated 12 mm coverslip. After 5–10 min, the cells will adhere to the bottom of the coverslip (Figure 2).
    **Figure 2.**
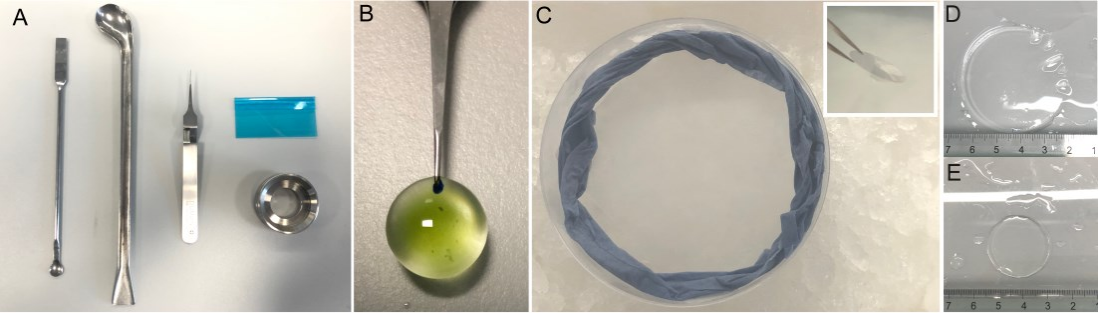
Tools and visual aids for *Chlamydomonas* ultrastructure expansion microscopy (U-ExM). A. Spatula, spoon, tweezers, plastic blade, and imaging chamber used in U-ExM. B. *Chlamydomonas* seeded on a 12 mm coverslip prior to fixation. The tweezer used to hold the coverslip here is a Tweezer Negative-Action Style no. 5. C. Gelation chamber on ice used to form gels. The blue ring is a dampened paper towel. Inset: 45° drop of coverslip onto gel droplet. D. Representative gel after the first round of expansion. E. Representative gel prior to expansion.
  2. Proceed to fixation steps.
    Multiple options for fixation are possible and should be chosen depending on the cellular compartment to be imaged. Fixation choices include cold methanol, paraformaldehyde + glutaraldehyde (PFA + GA), cryo-fixing by plunge freezing (Laporte et al., 2022b), and our recently optimized cryo-fixation protocol for ideal membrane preservation, by using 0.1% PFA + 0.02% GA in acetone during the substitution procedure (Louvel et al., 2022). Methanol fixation is well known to preserve the cytoskeletal network but generates a large amount of cytoplasmic and membrane clearance of other cellular components due to protein precipitation and extraction. On the other hand, PFA + GA is a gold standard fixation method to preserve membrane-bound organelles, such as mitochondria and the Golgi apparatus, but results in a poor expansion of some elements of the cytoskeleton. In particular, we observed that the centriole does not expand well if the fixation contains too much GA, whereas PFA or FA do not seem to interfere with the expansion factor (Gambarotto et al., 2019). Finally, cryo-fixation followed by freeze-substitution, recently adapted for immunofluorescence and expansion microscopy techniques, is the method of choice to best preserve cells in their native state (Laporte et al., 2022b). This method coupled with U-ExM has been shown to preserve the cytoskeleton as well as membranous structures. To further improve membrane preservation, addition of PFA + GA in the freeze-substitution step was recently adapted (Louvel et al., 2022) (Figure 3). It is to be noted that cells can also remain unfixed, especially to visualize the basal bodies and flagella, which remain intact under this condition, as well as some fibrous structures as visualized by Centrin staining (Figure 3A–3C) (van den Hoek et al., 2022). However, some cellular structures might be affected, such as dynamic microtubules (Gambarotto et al., 2021), membranous organelles, or the pyrenoid (Figure 3B). To best preserve the organization of these organelles, cryo-fixation is then the approach of choice (Laporte et al., 2022b; Louvel et al., 2022) (Figure 3D).
    **Figure 3.**
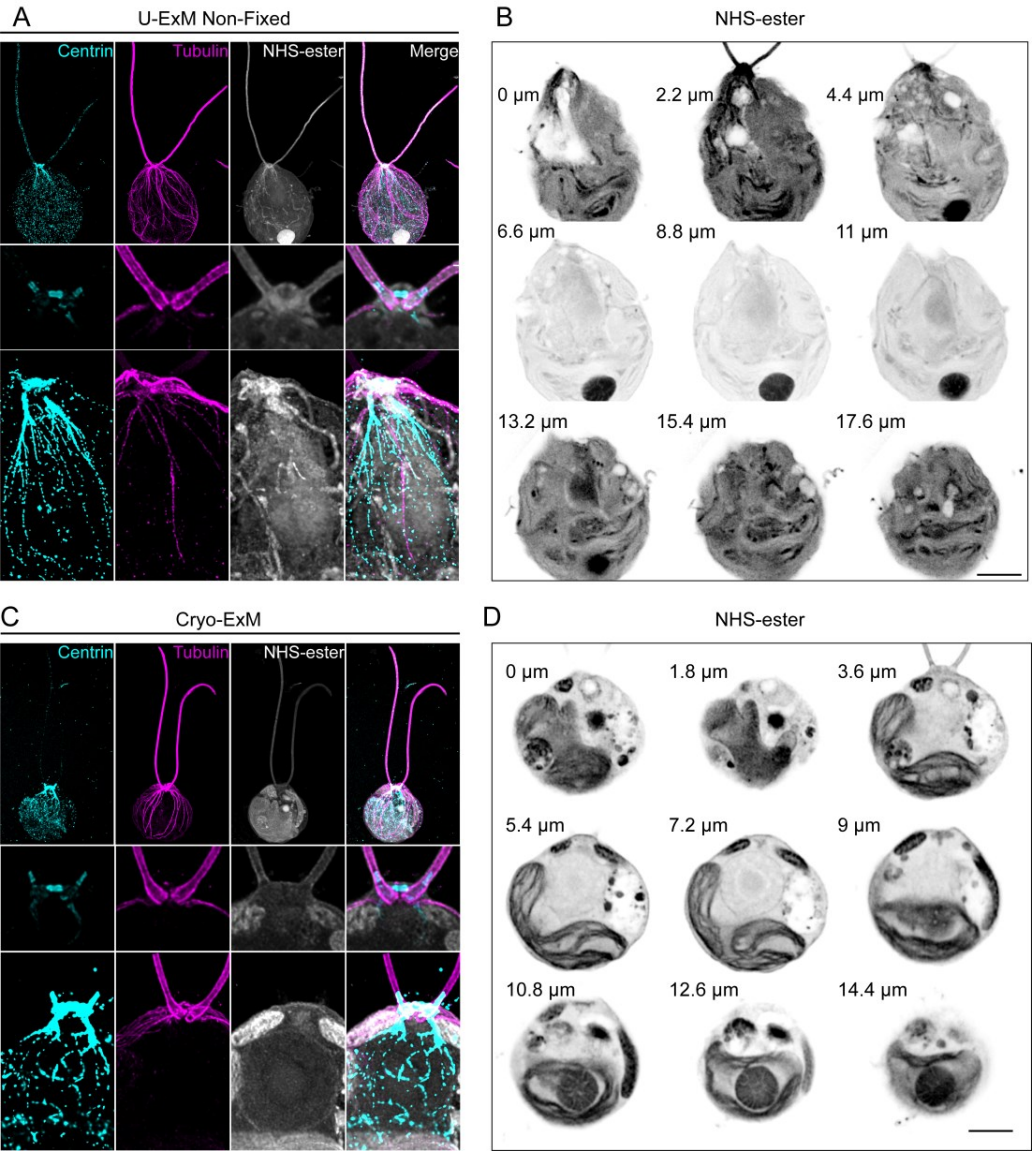
Comparison of ultrastructure expansion microscopy (U-ExM) without fixation and cryo-ExM. A. *Chlamydomonas* CW15- cells were expanded following the U-ExM protocol. No fixation was performed prior to expansion. Gels were stained with Centrin (cyan), Tubulin (magenta), and NHS-ester (gray). Top panel shows the whole cell. Note that the Centrin signal was overexposed to unveil the Centrin localization around the nucleus. Middle panel shows the inner basal body and striated fiber localization of Centrin. Bottom panel shows Centrin localization at NBBC (nucleus-basal body connector). Images were acquired with a Leica TCS SP8. Scale bar: 10 μm. The scale bar shows the measured physical length that was not rescaled based on the expansion factor. B. Grouped maximum intensity projection of 10 z-stacks from the same cell as in (A). Nine grouped projects were montaged together using ImageJ montage stacks tool. Numbers left to the cells indicate the physical distance from z = 0. Images were acquired with Leica TCS SP8 with a z-step size of 0.22 μm. Scale bar: 10 μm. The scale bar shows the measured physical length that was not rescaled based on the expansion factor. C. *Chlamydomonas* CW15- cells were expanded following the cryo-ExM protocol. Gels were stained with Centrin (cyan), Tubulin (magenta), and NHS-ester (gray). Top panel shows the whole cell. Note that the Centrin signal was overexposed to unveil the Centrin localization around the nucleus. Middle panel illustrates the inner basal body and striated fiber localization of Centrin. Bottom panel highlights Centrin localization at NBBC. Images were acquired with Leica TCS SP8. Scale bar: 10 μm. The scale bar shows the measured physical length that was not rescaled based on expansion factor. D. Grouped maximum intensity projection of 10 z-stacks from the same cell as in (C). Nine grouped projects were montaged together using ImageJ montage stacks tool. Numbers left to the cells indicate the physical distance from z = 0. Images were acquired with Leica TCS SP8 with a z-step size of 0.18 μm. Scale bar: 10 μm. The scale bar shows the measured physical length that was not rescaled based on the expansion factor.
**The different fixation procedures are as follows:**
    - Methanol
      Chill MeOH at -20 °C for at least 1 h. We usually store it at -20 °C in a little plastic box in which we immerse a small ceramic coverslip rack adapted for 12 mm coverslips, as described here (Guichard et al., 2015). Add 100 μL of *Chlamydomonas* culture to 12 mm PDL-coated coverslips. Incubate for 5 min, gently blot away liquid, and quickly place in the coverslip rack immersed in the cold MeOH. Place the container at -20 °C for 10 min. Remove coverslips and place in PBS. Store at 4 °C or process for U-ExM cross-linking prevention.
    - Cryo-ExM
      To perform cryo-fixation, first, fill a polystyrene box with dry ice and prepare 5 mL Eppendorf tubes with 2.5 mL of acetone (one tube per condition/coverslip). Snap-freeze the 5 mL tubes in liquid nitrogen. Next, fill a cryo-plunging station (Figure 4A) with liquid nitrogen to cool to -180 °C (approximately 5 min) and add ethane or an ethane-propane mixture to the cryogen container. When the plunging station is prepared, add 100 μL of *Chlamydomonas* culture to 12 mm PDL-coated coverslips (Figure 2B). Incubate for 5 min and use the plunging tweezers to grab the coverslip at approximately halfway through the coverslip (Figure 4B). Blot the coverslip by applying a piece of filter paper to its bottom for approximately 3 s. There will be an initial excess of media absorbed by the filter paper but allow for a thin layer of media to remain on the coverslip. Plunging stations contain a button or foot pedal to initiate the rapid plunging of the coverslip into the cryogen. Press the button or foot pedal to plunge the coverslip immediately after blotting (Figure 4C). The coverslip is then transferred to solid acetone to proceed to the freeze-substitution step. Note that this transfer must be done in the ethane gas area above the liquid nitrogen to prevent the coverslip from heating up and losing the vitreous state.
      To do so, rapidly transfer the plunge frozen coverslip into the 5 mL tube containing frozen acetone (Figure 4D). Place the 5 mL tubes at a 45° angle, immersed in dry ice. Agitate gently overnight using a tabletop shaker (50 rpm), allowing the temperature to rise to -80  °C. The following morning, remove the dry ice to allow the samples to reach 0 °C, which takes approximately 1.5 h. After reaching 0 °C, measured using a thermometer, use a 12-well plate to perform successive 5 min ethanol:water baths in order to rehydrate. The order is as follows: 100% EtOH, 100% EtOH, 95% EtOH, 95% EtOH, 70% EtOH, 50% EtOH, and lastly, PBS. The coverslips can now be processed for U-ExM cross-linking prevention.
      **Figure 4.**
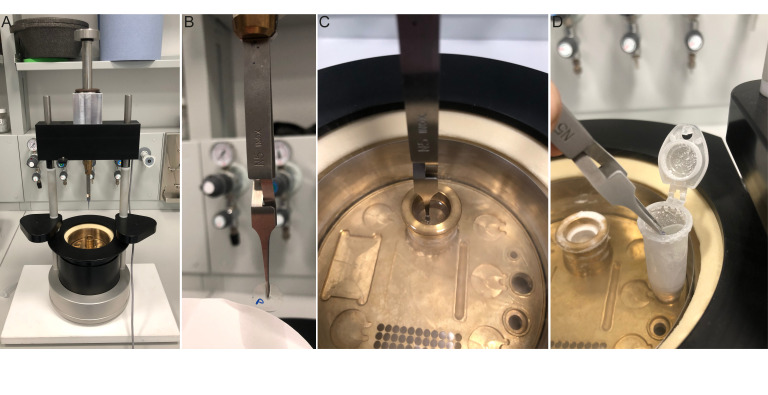
The setup and methodology of 12 mm coverslip cryo-fixation. A. Manual plunger for rapid vitrification in liquid ethane. B. Plunging tweezers holding cell-containing coverslip while blotting with filter paper. Note the position of the tweezers halfway down the coverslip. P denotes PDL and cell containing side. C. Plunged coverslip immersed in the liquid ethane of the cryogenic chamber. D. Representative transfer of the coverslip from the cryogenic chamber to the 5 mL tube containing frozen acetone. The tube is opened to facilitate the rapid transfer of the coverslip, and transfer occurs in the ethane gas area, just above the liquid nitrogen.
    - Membrane optimized cryo-ExM
      To better preserve the membranes and retain the lipids, the freeze-substitution step can be done as follows (Louvel et al., 2022). Prior to plunge freezing, make a mixture of 0.1% PFA + 0.02% GA in acetone in a 5 mL Eppendorf tube and put the tube in the liquid nitrogen to cool it, resulting in solid acetone. The tube should stay vertical during this step. Then, proceed for cryo-fixation using plunge-freezing as explained above. For the substitution step, coverslips are transferred into the 5 mL Eppendorf tube containing solid acetone supplemented with PFA and GA. As for the initial cryo-ExM protocol, place the 5 mL tubes at a 45° angle, emersed in dry ice. Agitate gently overnight, allowing the temperature to rise to -80  °C. The next morning, remove the remaining dry ice to bring the temperature up to approximately 0–4 °C. Next, rehydrate the samples by incubating the coverslips in successive 4 °C ethanol baths also containing 0.1% PFA + 0.02% GA. The order is as follows: ethanol 100% + 0.1% PFA + 0.02% GA (5 min), ethanol 100% + 0.1% PFA + 0.02% GA (5 min), ethanol 95% + 0.1% PFA + 0.02% GA (3 min), ethanol 95% + 0.1% PFA + 0.02% GA (3 min), ethanol 70% + 0.1% PFA + 0.02% GA (3 min), ethanol 50% + 0.1% PFA + 0.02% GA (3 min), ethanol 25% + 0.1% PFA + 0.02% GA (3 min), water (3 min), and PBS.
*Note: We have also observed that the 70%, 50%, and 25% ethanol steps can be performed without the addition of PFA + GA, and membranes remain preserved, as shown in Figure 5.*
    - Unfixed
      Add 100 μL of *Chlamydomonas* culture to 12 mm PDL-coated coverslips and proceed directly to U-ExM cross-linking prevention step.
  3. After fixation, the protocol proceeds as follows. For each fixed coverslip, make a crosslinking prevention solution containing 2.0% AA/1.4% FA in PBS in a total volume of 1 mL per sample. Add individual coverslips to wells of a 12-well plate and add 1 mL of crosslinking prevention solution. Fill unused wells with ddH_2_O to prevent evaporation, wrap the edge of the plate with parafilm, and incubate at 37 °C for 3 h.
  4. When the coverslips have been incubating for approximately 3 h, place a gelation chamber (Figure 2) at 4 °C to chill. Place the required number of monomer solution (MS) aliquots (one aliquot per two coverslips), as well as an aliquot of 10% APS and 10% TEMED, on ice.
*Note: Aliquots of APS and TEMED can be stored again for future use, while the MS is for one-time use.*
  5. The gelation chamber is created by simply placing a damp paper towel to create humidity into a polystyrene box or dish on top of a piece of parafilm (Figure 2C).
  6. Retrieve the samples from crosslinking prevention and remove coverslips from the well. Gently blot away excess liquid and place the chilled humidity chamber into an ice bucket as leveled as possible (Figure 2). Pre-set a P200 pipette to 35 μL and a P20 pipette to 5 μL.
  7. Once the gelation chamber is prepared, make the activated MS by adding 5 μL of APS and then 5 μL of TEMED to the 90 μL MS aliquot. Rapidly, yet calmly, vortex the activated solution and add two 35 μL droplets to the parafilm on the bottom of the humidity chamber (Figure 2). Quickly place the two coverslips, one by one, cell-side facing down, on the droplet with the coverslip taking a 45° angle (Figure 2, inset).
  8. Let the gelation chamber sit for 5 min on ice and then transfer it to a 37 °C environment for 1 h.
  9. Retrieve the gelation chamber from 37 °C and remove the now formed gel associated with the coverslip with a spatula. Place the gel/coverslip associated–side face down, in the well of a 6-well plate. Use a Pasteur pipette to add approximately 1 mL of denaturation buffer to each sample. Place the 6-well plate on a shaker for 15 min at RT. At this stage, the gel will start to expand approximately 2× and dissociate from the coverslip.
  10. Prewarm a heat block to 95 °C and add 1 mL of denaturation solution to 1.5 mL Eppendorf tubes, one per sample. After gels have dissociated from the coverslips, use a spoon to collect the gels and place them in Eppendorf tubes filled with denaturation buffer (gels are slightly rolled to enter in the tube). Make sure that the gels are completely submerged in denaturation buffer and add Eppendorf tubes to the 95 °C heat block for 1.5 h. Place a weight on top of the tubes to prevent them from popping.
  11. After the 1.5 h of denaturation, remove the denaturation solution into a waste solution using a spatula to trap the gel at the edge of Eppendorf tube. Immediately pour the gel into a 500 mL beaker containing approximately 100 mL of ddH_2_O. Let the gel wash for 30 min and remove the ddH_2_O. Perform one more wash as just described, then refill with ddH_2_O and let the gel sit overnight for the first round of expansion. *Note: When removing the water during gel washes, a fine-mesh net can be used to cover the waste beaker to catch the gel if it falls.*
- **Antibody staining of gels (post-expansion labeling) (day 1)**
  1. Prepare stocks of PBS-0.1% Tween and PBS-2% BSA and store at 4 °C.
  2. Remove the gel from the 500 mL flask and place it on a clean, flat surface. Ensure that the gel does not completely dry. Use a caliper to measure the diameter of the fully expanded gel to determine the expansion factor. From a 12 mm coverslip, a measured expanded gel diameter of 54 mm corresponds to an expansion factor of 4.5 (Figure 2).
  3. Shrink the gels by placing them into 500 mL beakers with 100 mL of PBS and let sit for 15 min. Repeat 1×.
  4. Place the shrunken gels on a flat surface, again ensuring that the gel remains slightly hydrated. Cut the gel into four quarter pieces with a razor blade and place each piece in the well of a 12-well plate.
*Note: It is possible to keep the entire gel for immunostaining, but a 6-well plate is then used with twice the volume of antibody (500 μL of total volume vs. 1 mL; next step of this section). Cutting the gel further enables more antibody staining conditions on the same sample. If not all gel pieces are needed for immediate staining, the pieces can be stored in PBS at 4 °C for a few weeks or in a 50% glycerol solution at -20 °C for longer (see Notes section).*
  5. Add the desired primary antibody combinations to a final volume of 500 μL of PBS-2% BSA and add this mixture to the desired gels. (Note that antibody concentration requires optimization, but a 1:300 dilution is a good place to start.)
  6. Seal the 12-well plate with parafilm and place it on a shaker at 37 °C for 3 h.
  7. Retrieve the 12-well plate and remove primary antibody solution from wells with a Pasteur pipette. Add approximately 1 mL of PBS-0.1% Tween to gels and gently shake at RT for 10 min. Repeat 2× to completely wash unbound antibodies.
  8. After completion of the PBS-0.1% Tween washes, prepare desired secondary antibody combinations in 500 μL of PBS 2% BSA. Add the secondary antibody solutions to the corresponding gel pieces and wrap the 12-well plate in aluminum foil, as the fluorophore of the secondaries may be photosensitive.
*Note: 1:400 dilution is a reasonable concentration for most secondary antibodies.*
  9. Place the wrapped 12-well plate on a shaker at 37 °C for 3 h.
  10. Retrieve the 12-well plate and repeat the PBS-0.1% Tween washes described in step D7. Take care to keep the 12-well plate wrapped during the washes.
  11. After completion of the final wash, proceed to either NHS-ester staining, if desired (section E), or re-expansion. For re-expansion, use a spoon or spatula to remove the gel piece from the well, and place it in a 250 mL beaker containing approximately 50 mL of ddH_2_O. Incubate at RT for 30 min and remove the water. Repeat one more time and then incubate gels in ddH_2_O until imaging.
*Note: NHS-ester staining can also be performed the next day. Keep gel pieces in a 12-well plate, add PBS to the well, and store at 4 °C until NHS-ester staining is performed.*
    **Table 1.** Antibodies against tagged proteins

Tag	Species	Manufacturer	Ref	U-ExM Dilution
GFP	rabbit	Torrey Pines	TP401	1:200
YFP	rabbit	Torrey Pines	TP401	1:200
mNeongreen*	mouse	Chromotek	32F6	Non compatible
HA	mouse	Sigma-Aldrich	H6908	1:100
HRV3C	rabbit	Thermo Fisher	PA1-118	1:150
SNAP	mouse	NEB	P9310S	1:100
    **Antibodies against tagged proteins.** List of antibodies against tags, the manufacturer, and dilutions tested in the U-ExM of tagged *Chlamydomonas* strains.
- **NHS-ester staining**
  NHS-ester staining in expansion microscopy has been introduced by M’Saad and Bewersdorf (M’Saad and Bewersdorf, 2020) as well as Mao et al. (2020) and Yu et al. (2020). NHS-ester is an ester-dye conjugate that reacts with primary amines on proteins. With this labeling, it is possible to reveal the cellular context, comparable to electron microscopy images (M’Saad and Bewersdorf, 2020).
  1. Prior to the experiments, make 20 μL working aliquots of NHS-ester dye (1 mg/mL) by suspension in PBS and store at -20 °C. NHS-ester dye is photosensitive. Store in a lightproof box. Note: NHS-ester can be conjugated against many different fluorophores, resulting in preferential labeling of different cellular compartments based on hydrophobicity, pH, etc. (Sim et al., 2021). This protocol describes the concentrations to use in NHS-ester conjugated against Atto 488, 405, 594, and 647.
  2. The day of the experiment, remove the desired number of working aliquots from the freezer and thaw in the dark.
  3. Add the thawed working aliquot to a final volume of 1 mL of PBS to generate a final concentration of 20 μg/mL NHS-ester. Add 500 μL of NHS-ester solution per well containing gels in PBS. Note: one working aliquot of 20 μL of NHS-ester at 1 mg/mL corresponds to staining two wells of a 12-well plate. Diluting further is also possible.
  4. Cover the 12-well plate in aluminum foil and incubate for 1.5 h at RT on a shaker.
  5. Remove the NHS-ester staining solution from wells with a Pasteur pipette and replace it with approximately 1 mL of PBS for washing. Place on the shaker for 10 min. Repeat the washes 4×.
  6. After the last wash, proceed to re-expansion in ddH_2_O as described in step D11.
- **BODIPY^TM^ membrane staining**
  In order to preserve the lipid membranes in U-ExM, cryo-fixation followed by freeze-substitution containing PFA and GA should be used to obtain an optimal result. To visualize the membranes, it is then possible to use BODIPY^TM^ in the same way as with NHS-ester (Figure 5).
  **Figure 5.**
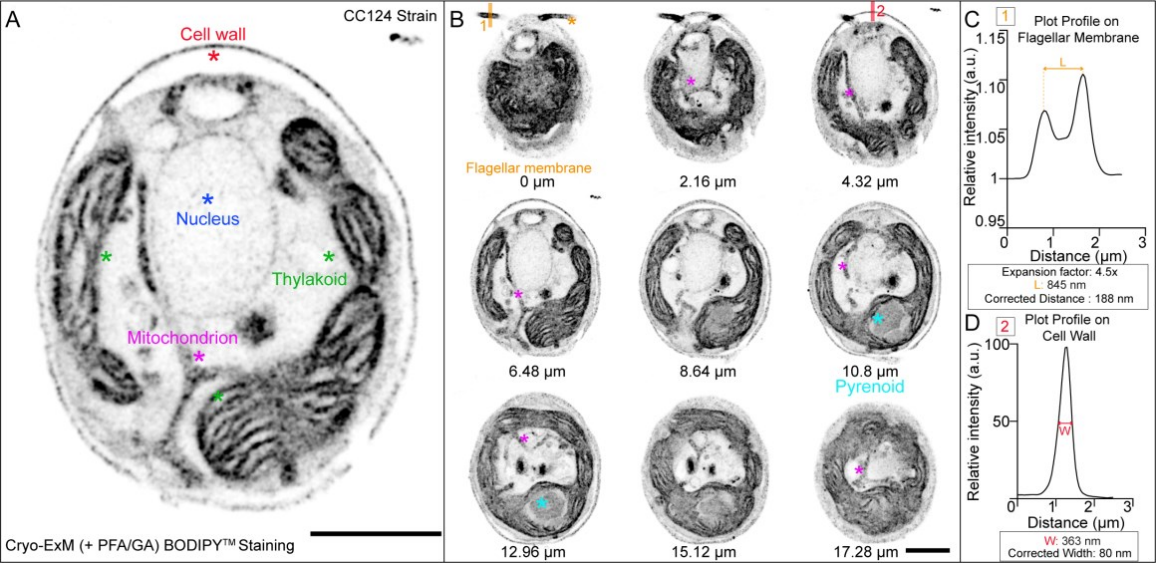
BODIPY^TM^ lipid staining of *Chlamydomonas* using membrane-optimized cryo-ExM protocol. A. *Chlamydomonas* CC124- strain was cryo-fixed and freeze-substituted in acetone supplemented with 0.1% PFA and 0.02% GA prior to expansion (see cryo-fixation step for detail). The gel was stained with BODIPY^TM^. The image shows a single z-plane. Observed structures are labeled with a star. Scale bar: 10 μm. The scale bar shows the measured physical length that was not rescaled based on the expansion factor. B. Grouped maximum intensity projection of 12 z-stacks from the same cell as in (A). Nine grouped projects were montaged together using ImageJ montage stacks tool. Note that the flagellar membrane and pyrenoid structures are shown here, which were not visible in the single z-plane shown in (A). Numbers under the cells indicate the physical distance from z = 0. Images were acquired with Leica TCS SP8 with a z-step size of 0.18 μm. Scale bar: 10 μm. The scale bar shows the measured physical length that was not rescaled based on the expansion factor. C. The plot profile is drawn on the flagellar membrane using ImageJ line scan tool [shown as 1 in (B)]. L shows the measured peak-to-peak distance. The measured length is corrected by the expansion factor. The corrected distance shows the diameter of the flagellar membrane based on BODIPY^TM^ staining. D. The plot profile is drawn on the cell wall using ImageJ line scan tool [shown as 2 in (B)]. W shows the distance between 50% intensity of the peak.
  1. Make 10 μL aliquots of BODIPY^TM^ at 2 mM by diluting in DMSO. Unused aliquots should be stored at -20 °C, away from the light.
  2. To stain for BODIPY^TM^, perform a 1:200 dilution with PBS 2% BSA and add to wells containing the shrunken gels in PBS. One aliquot of 10 μL of BODIPY^TM^ should be enough to stain two pieces of gel in the 12-well plate.
  3. Cover the 12-well plate in aluminum foil and incubate for 1.5–2 h at RT on a shaker.
  4. Remove the BODIPY^TM^ staining solution from wells with a Pasteur pipette and replace with approximately 1 mL of PBS for washing. Place on the shaker for 10 min. Repeat the washes 3×.
  5. After the last wash, proceed to re-expansion in ddH_2_O as described in step D11.
- **Imaging of gels (days 2–7+)**
  1. Remove the expanded gel piece from the 250 mL beaker by pouring out the water while trapping the piece (with a spatula or manually by wearing a glove). Place it on a clean and flat surface and cut a square piece for imaging that is approximately 1.5 cm × 1.5 cm in size. Return the remaining gel pieces to the beaker containing ddH_2_O for potential future use (see Note 5).
  2. Place the 1.5 cm × 1.5 cm gel piece in the center of a 24 mm coverslip and place the coverslip inside of the imaging chamber (Imaging chamber example, Figure 2A).
*Note: The gel piece should not touch the edges of the imaging chamber to prevent drifting during acquisition.*
  3. Make sure an appropriate stage mount is present on the microscope stage and insert the imaging chamber.
  4. Change the objective to medium magnification (20× or similar) to determine the side of the gel containing the cells.
*Note: This may require removing the imaging chamber and flipping the gel piece with a spatula. If the gel is sided inversely, heavy background fluorescence can be observed in the imaging plane.*
  5. When the correct sidedness of the gel is determined, gently blot the four edges of the gel as well as the reverse side to remove excess water. Retrieve a PDL-coated 24 mm coverslip and place the cell-containing side face down in the center. Use a small paintbrush or the side of a spatula to gently mount the gel against the PDL coating, until the gel is completely adherent to the surface. Place the PDL-coated coverslip containing the gel back into the imaging disk. Gently add approximately 5 μL of ddH_2_O to the top of the gel and place a 24 mm coverslip on top to create a mini humid chamber.
*Note: Failure to remove excess liquid of the gel or poor PDL coating results in substantial sample drift during acquisition and failure to collect images. See Notes section for assistance with sample drift.*
  6. Return the imaging chamber to the microscope stage and perform acquisition, adding 5 μL of ddH_2_O approximately every hour to prevent gel shrinkage. Add a non-coated 24 mm coverslip to the top of the chamber to prevent water evaporation.
  7. *Chlamydomonas* cells are positioned in different orientations within the gel. Examine the gel to find a sample in the desired orientation.
  8. When imaging with a Leica microscope, image in Lightning mode, with the strategy as “Adaptive” and “Water” selected as the mounting medium for the best results. If using a Leica confocal such as an SP8, set the pixel size to 35 nm for maximum resolution. If imaging with a Zeiss microscope such as an LSM980, image in Airyscan mode with “Resolution” selected. (Representative z-stack, Video 1.)
*Note: Acquiring a pixel size of 35 nm is the maximum boundary when Nyquist sampling, owing to the expected lateral resolution size of 70 nm as previously published (Chen et al., 2015). However, given that the optical properties of the microscope are unchanged, sampling with a larger pixel size will decrease acquisition time with minimal impact on the resolution. See data analysis for a more in-depth explanation of the resolution.*
    **Video 1.**
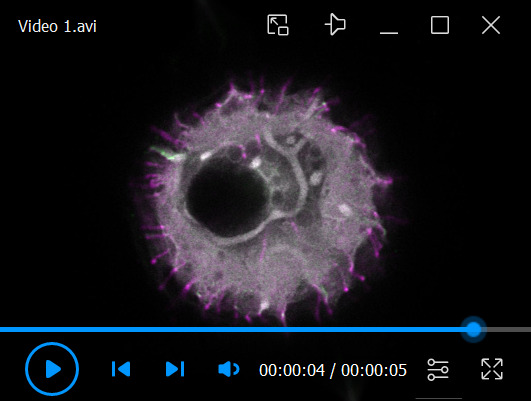
Representative z-stack of a top view of an expanded *Chlamydomonas* cell. Cryo-ExM of a transgenic cell expressing IFT46-mNeonGreen:D1bLIC-mCherry imaged with a LSM980 confocal microscope using Airyscan mode with a step size of 0.15 μm. The staining was performed with NHS-ester (gray), anti-Tubulin antibody (magenta), and anti-Acetylated Tubulin antibody (green). Scale bar corrected by the expansion factor: 5 μm.

## Data analysis

Traditional application of confocal microscopy is conservatively diffraction-limited to approximately 200 nm in the lateral dimensions (MacDonald et al., 2015). A normal expansion factor of 4.2–4.5× yields an effective lateral resolution of approximately 70 nm as measured in the original expansion microscopy protocol (Chen et al., 2015). With this resolution, it is possible to resolve the membranes, labeled with BODIPY, on either side of the flagella with a distance of 188 nm (Figure 5C). Furthermore, when measuring the thickness of the cell wall, which is also stained by BODIPY, the 40 nm thickness of the cell wall (Horne et al., 1971) appears as an 80 nm thick layer. This is consistent with a resolution of approximately 70 nm.

To measure the isotropy of an expanded sample, features observable in non-expanded *Chlamydomonas* can be measured post-fixation, but prior to starting cross-linking prevention of U-ExM. For example, the nucleus size, pyrenoid area, or flagellar length can be measured by brightfield microscopy and then re-measured after U-ExM to confirm isotropy.

When analyzing collected images, it is necessary to know the expansion factor to properly scale distances. As mentioned in Section D, the expansion factor can be used to scale images. In our laboratories, we also routinely use a well described benchmark, such as the width of a basal body, to scale images. For example, a basal body with an expanded proximal width of 900 nm corresponds to an expansion factor of 4, given the 225 actual width of the basal body. Otherwise, we found in U-ExM that measuring the gel before and after expansion is quite reliable to determine the expansion factor, with values similar to those obtained by measuring the basal body, so this simpler approach can be used if you do not have an internal molecular ruler.

**Figure 6.**
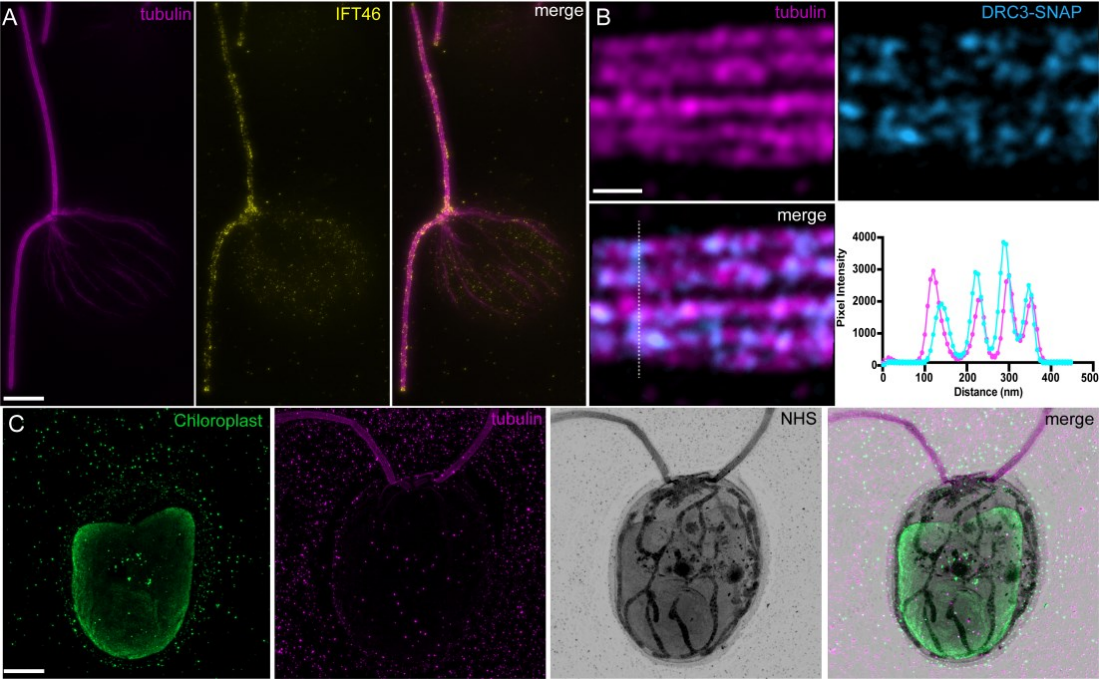
Representative examples of *Chlamydomonas* imaging in ultrastructure expansion microscopy (U-ExM). A. Methanol-fixed *Chlamydomonas* IFT46-YFP cell stained for tubulin and IFT46. Note the accumulation of intraflagellar transport trains (IFT) particles at the basal body and flagellar tip, as well as train-like staining on the flagellar axoneme. Scale bar corrected by the expansion factor: 2 μm. B. High-magnification image of tubulin staining with SNAP-tagged DRC3, a component of the dynein regulatory complex. Bottom right panel is a plot profile generated from the dotted line in the DRC3 staining. Scale bar corrected by the expansion factor: 50 nm. C. Cryo-ExM-fixed *Chlamydomonas* cell stained for the chloroplast (PsbA staining), tubulin, and NHS-ester. Scale bar corrected by the expansion factor: 2 μm. Images were acquired with a Leica Thunder (A) and LSM980 Airyscan2 (B, C).

A broad range of cellular compartments can be visualized through antibody labeling, for example IFT train localization at the basal body and along the flagella by antibody labeling against an IFT component (Figure 6A), contrasted against the labeling of the chloroplast in the cell body (Figure 6C, Video 2).

To perform analyses, any regular image analysis software can be used. We routinely use Fiji, in particular the plot profile function to assess colocalization and measure the distances and periodicities along a substrate, using peak-to-peak or full-width half-maximum. For instance, we stained for the Dynein regulatory complex subunit 3 (DRC3), a flagellar protein important for the regulation of the flagellar waveform (Huang et al., 1982), and found a colocalization signal with the microtubule doublets (Figure 6B).

**Video 2.**
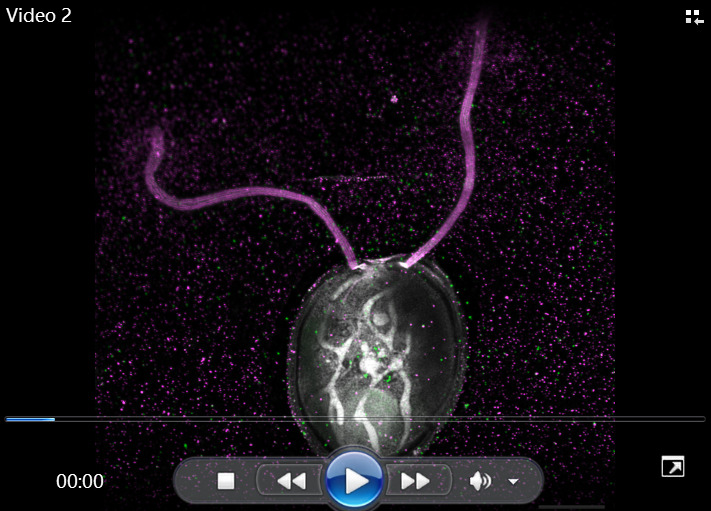
Representative z-stack of an expanded *Chlamydomonas* cell with chloroplast staining. Cryo-ExM of a transgenic cell expressing IFT46-mNeonGreen:D1bLIC-mCherry imaged with a LSM980-NLO confocal microscope using Airyscan mode with a step size of 0.15 μm. The staining was performed with NHS-ester (gray), anti-acetylated Tubulin antibody (magenta), and anti-PsbA antibody (green). Scale bar corrected by the expansion factor: 10 μm.

## Notes

1. Sodium acrylate
  As with all expansion microscopy protocols, the quality of sodium acrylate is a frequent cause of expansion microscopy issues. Dissolving the sodium acrylate powder gradually into stirring water facilitates dissolution. Take care to dissolve the sodium acrylate on ice at 4 °C, or immediately transfer to 4 °C. Different sodium acrylate batches are variable in purity. Only solutions that are clear or faintly yellow should be used in experiments.
2. Fixation methods
  The desired fixation method should be considered prior to starting the experiment. Each method has its benefits and detractions, but cryo-fixation offers the best preservation of samples in their native state (Dubochet et al., 1988). If cryo-fixation is unavailable, consider the cellular compartment to be stained, and proceed accordingly with either unfixed, methanol, or PFA/GA fixations.
  When fixing, especially if using cryo-fixation, it is important to make sure that the coverslip is not overly blotted. Completely drying the coverslip will result in dehydrated cells where the cytoplasmic volume shrinks but the cell wall remains at the same volume, resulting in a large gap between the cytoplasmic volume and cell wall, creating the appearance of a “halo” in expansion microscopy. Avoiding over-blotting of the coverslip circumvents this artifact.
3. Broken flagella while imaging
  *Chlamydomonas* often adheres to surfaces via their flagella. In these cases, the flagella may fragment during expansion, and cracks or pieces can be observed on the coverslip. This is common, and the gel can be scanned to find intact flagella.
4. Microscopic drift
  As stated in the Procedure section, sample drift at the microscope is a common problem, resulting in failure to acquire usable images. We found that plasma-cleaning the surface of 24 mm coverslips prior to the addition of PDL greatly enhances the adhesion between gel and coverslip. The gel should remain completely adherent even after the addition of the water to prevent gel shrinkage. Avoid imaging at the edges of the gels and stay towards the center for data collection. If the gels are noticeably drifting at the initiation of imaging, remove the imaging chamber from the microscope, gently blot the edges of the gel, and return to the microscope for imaging. Freshly PDL-coated coverslips result in less drift.
5. Gel storage
  The imaging quality of a gel decreases over time due to progressive fluorescence degradation or antibody loss. We recommend imaging the gels as close to the time of staining as possible. Imaging up to seven days post-staining is generally permissible once they have been expanded in ddH_2_O. Longer storage of both labeled and unlabeled gels can be attained by immersing expanded gels in ddH_2_O containing 50% glycerol in a 10 cm dish, shaking for 3 h, wrapping the dish in parafilm, and placing at -20 °C. To thaw the frozen gels, wash once with ddH_2_O to remove the excess glycerol and then wash at least three times in PBS (1×) for 1 h. (The gel will shrink in PBS and force the glycerol out.) Make sure to wash off the glycerol completely or a signal background might be induced in the staining.

## Recipes

All stocks are assumed to be the same as those listed in reagents.

1. **TAP, 0.5 L**
  TRIS, 1.21 g
  Phosphate buffer II, 0.5 mL
  Solution A, 5 mL
  Hutner’s trace elements, 0.5 mL
  Acetic acid, 0.5 mL
  Mix the ingredients and add water to bring to 0.5 L. Adjust pH to 7.0. Autoclave to sterilize.
2. **Phosphate buffer II**
  1 M K_2_HPO_4_, 250 mL
  1 M KH_2_PO_4_, 170 mL
  Adjust the pH to 7
3. **Solution A (40×)**
  Tris, 96.8 g
  Phosphate buffer (pH 7), 40 mL
  Acetic acid, 40 mL
  Adjust the solution to 1L with distilled water.
4. **Formaldehyde/acrylamide mixture (1.4% FA, 2% AA), 1 mL/1 coverslip**
  FA (38%), 38 μL
  AA (40%), 50 μL
  PBS 1×, 912 μL
  Mix ingredients and add 1 mL to a well of a 12-well plate. The table below displays the volume of each reagent when scaling up for number of coverslips.
  **Table:** 

Coverslips	1	2	3	4	5	6	7	8
**FA (1.4%)**	38	76	114	152	190	228	266	304
**AA (2.0%)**	50	100	150	200	250	300	350	400
**PBS (1×)**	912	1824	2736	3648	4560	5472	6384	7296
5. **Sodium acrylate solution (38% w/w)**
  Sodium acrylate, 19 g
  Nuclease-free water, 31 mL*^!^
  Add the sodium acrylate incrementally in stirring water to mix. Attempt to work at 4 °C and store at 4 °C immediately after sodium acrylate is dissolved.
  *31 mL of nuclease-free water = 31 g of nuclease-free water. 31 g of nuclease-free water + 19 g of sodium acrylate = 50 g total. 19 g of sodium acrylate/50 g total = 38% w/w solution.
  !38% w/w solution of sodium acrylate corresponds to a 46% w/v solution owing to the high density of sodium acrylate in solution.
6. Monomer solution, 10 aliquots of 90 μL*
  Sodium acrylate solution (38% w/w), 500 μL
  Acrylamide (40%), 250 μL
  BIS (2%), 50 μL
  10× PBS, 100 μL
  Mix the ingredients on ice and vortex for 30 s. Make 90 μL aliquots and store at -20 °C for three weeks. One aliquot is used for two coverslips.
7. **10% TEMED, 10 aliquots of 100 μL**
  TEMED, 100 μL
  Nuclease-free water, 900 μL
  Mix TEMED and nuclease-free water. Make 100 μL aliquots and store at -20 °C.
8. **10% APS, 10 aliquots of 100 μL**
  APS, 0.1 g
  Nuclease-free water, 1,000 μL
  Mix APS powder and nuclease-free water. Make 100 μL aliquots and store at -20 °C.
9. **Denaturation buffer, pH 9, 100 mL**
  SDS solution (350 mM stock solution, 10 g in ddH_2_O, 100 mL final volume), 57.14 mL
  NaCl (5 M), 4 mL
  Tris-BASE, 0.6 g
  ddH_2_O, fill to 100 mL
  Dissolve 0.6 g of TRIS-BASE in 10 mL of ddH_2_O. Add NaCl and SDS. Adjust to pH 9 with HCl. Fill to 100 mL with ddH_2_O and store at RT.
10. **PBS-Tween (0.1% w/v) (1×, 1 L)**
  Prepare 1× PBS:
  NaCl, 8 g
  KCL, 0.2 g
  Na_2_HPO_4_,1.44 g
  KH_2_PO_4_, 0.24 g
  Adjust the solution to 1 L with distilled water.
  Add 100 µL Tween 20 detergent.
11. **PBS-BSA (2%) (1×, 100 mL)**
  Dissolve 2 g of BSA in 100 mL of 1× PBS.
